# Retro nasal blockade reduces the neural processing of sucrose in the human brain

**DOI:** 10.1016/j.ibneur.2025.10.020

**Published:** 2025-10-31

**Authors:** Hee-kyoung Ko, Jingang Shi, Thomas Eidenberger, Weiyao Shi, Ciara McCabe

**Affiliations:** a School of Psychology and Clinical Language Sciences, University of Reading, Reading, UK; b EPC Natural Products Co., Ltd. BDA, Building 1, 35 Jinghai 3rd Road, Beijing 101111, China; c University of Applied Sciences Upper Austria, Roseggerstrasse 15, Wels 4600, Austria

**Keywords:** Sucrose, Retro nasal, Nose clip, Olfaction, Neural

## Abstract

It is assumed “Non-volatile” tastes like sucrose do not activate retro nasal pathways. Recent studies find that sucrose when aerosolized, can reach the retro nasal olfactory region and be perceived. The neural mechanisms by which the human brain interprets sucrose via retro nasal pathways is unknown.

We examined neural activity to sucrose with a nose clip on (blocking retro nasal) and nose clip off, in healthy adults (N = 34, mean 25 yrs.). We examined the whole brain and ROIs involved in taste, smell, attention, reward and multi-modal integration; insula, postcentral gyrus, amygdala, olfactory cortex, subgenual and pregenual anterior cingulate, nucleus accumbens and OFC. We also examined correlations with subjective ratings of pleasantness and mouth fullness.

The nose clip on vs off reduced the subjective experience of mouth fullness. Neural activity to sucrose was reduced with the nose clip on in the primary taste, olfactory, attention and reward ROIs and in the rolandic operculum, lingual gyrus and precuneus in the whole brain analyses. The olfactory and prefrontal cortex ROIs tracked subjective mouth fullness, but this was not apparent with the nose clip on.

Blocking retro nasal sensation reduces subjective and neural responses to sucrose taste. Retro nasal sensations could play a role in “pure” taste perception. Developing more satisfying low-sugar foods could be achieved by enhancing the perception of sweetness through aroma modulation.

## Introduction

Volatile odour molecules released from food or drink in the mouth travel up the back of the throat into the nasal cavity and activate the olfactory receptors via retro nasal pathways (Stevens and Cain, 1986, Voirol and Daget, 1986, Buettner et al., 2001). Sweetness perception begins in the oral cavity, where taste receptor cells are dedicated to sweet-sensing interact with sugars, artificial sweeteners, and other sweet-tasting chemicals (Juen, Lu et al. 2025). Sucrose is considered non-volatile and therefore not recognised by the retro nasal pathways (Roper and Chaudhari, 2017).

Yet disabling retro nasal sensation through reversed nasal airflow significantly impaired participants’ ability to identify sucrose, although most were still able to perceive its sweetness (Mozell, Smith et al. 1969). Similarly studies have reported that blocking retro nasal sensation with a nose clip increases detection and recognition thresholds (Murphy and Cain, 1980), reduces identification accuracy (Masaoka, Satoh et al. 2010), and diminishes the perceived sweetness intensity of sucrose solutions (Mojet et al., 2003, Mu et al., 2024, Yang et al., 2024). A nose clip can effect taste and olfactory senses but not other senses such as vision or touch, suggesting an important olfactory component even with “non-volatile” tastes (Yang, Kim et al. 2024).

The observed differences in sucrose sweetness perception in young people vs old diminishes when youth are wearing a nose clip, suggesting retro nasal sensations account for the differences (Mojet, Köster et al. 2005). Nasal blockage via sinusitis can also significantly reduce sucrose detection (Tsuji, Tanaka et al. 2018). Hence everyday colds could affect taste perception via retro nasal blockade (James, Palte et al. 2022) and viruses such as COVID19 could also impact taste perception via retro nasal dysfunction. As this can have serious psychological implications (Javed, Ijaz et al. 2022) it is imperative to understand the contribution of retro nasal pathways to taste processing.

Given sucrose is considered non-volatile some suggest it is impurities in taste solutions rather than the tastants themselves that is being recognised by retro nasal processing (Mojet, Köster et al. 2005). Others refute this as they observed that the taste purity grade (e.g., reagent grade, non-reagent grade, and food grade) did not influence olfactory discrimination, both in mice (Zukerman, Touzani et al. 2009) and humans (Chen, 2013).

One explanation for reduced perception with nasal blockage could be that non-volatile compounds such as sucrose taste do indeed activate the retro nasal pathways. Our recent study using high speed cameras found that an orally-ingested sucrose solution could be transferred to the nasal cavity in the form of aerosol particles (He, Chen et al. 2023). This plausibly explains how retro nasal sensation is involved in the oral consumption of non-volatile sucrose, affecting its identification, intensity perception and threshold detection. As sucrose sweetness intensity was reduced when the volunteers’ noses were clipped, this also indicates the involvement of retro nasal sensation during its drinking (He, Chen et al. 2023). These findings were extended with our findings that retro nasal sensation can contribute to the discrimination between tastes such as sucrose and sucralose and to the perception of sweeteners (He, Chen et al. 2024).

While these findings clearly highlight the involvement of retro nasal sensation in the perception of sucrose, the underlying neural mechanisms underpinning the involvement of the retro nasal pathway vs the ortho nasal pathway in sucrose taste perception is unknown.

Taste processing begins on the tongue and taste receptors. Next information is transmitted via sensory afferent fibres to brain areas involved in taste perception (Lee and Owyang, 2019). Functional magnetic resonance imaging (fMRI) reveals that taste activates the anterior insula/frontal operculum, the primary taste cortex, the orbitofrontal cortex (OFC) (possibly secondary taste cortex) and the anterior cingulate (ACC) (Rolls, 2019). Further, taste intensity correlates with activity in the insula whereas pleasantness correlates with prefrontal regions such as the OFC and ACC (Rolls, 2019).

Retro nasal processing allows molecules to reach the olfactory epithelium in the nasal cavity, where they bind to olfactory receptors. The receptors send electrical signals via the olfactory nerve to the olfactory bulb, located at the base of the brain. From here the signal is relayed to higher brain areas such as the piriform cortex, the primary area for odour perception. In humans, the piriform cortex is correlated with the intensity of odours but not their pleasantness (Rolls, 2019). Signals from the olfactory bulb also project to the OFC where odour and other visual and sensory information are combined to contribute to stimuli identification and evaluation (Rolls, 2015, Rolls, 2019). However, if non-volatile taste compounds such as sucrose are also perceived through retro nasal pathways, it raises the question of whether the blockade of retro nasal sensation would reduce or slow down the integration of neural responses to sucrose.

Sucrose is known for its sweetness but also for mouth fullness (Lavin, French et al. 2002) and nasal occlusion can diminish the perception of fullness (Baraniuk, 2011, Yeomans and Boakes, 2016) however neural activity underpinning sucrose pleasantness and fullness during nasal occlusion remains unexplored.

Therefore, the aim of this study was to examine the brains response to sucrose with and without a nose clip to block retro nasal processing. We examined whole brain activity and regions of interest in taste, olfactory and multi modal areas. We also examined the correlation between brain activity and subjective ratings of pleasantness and mouth fullness to see if this was impacted by retro nasal processing.

## Materials and methods

### Participants

Thirty-four healthy right-handed adults were recruited between 18 and 45 years old with healthy weights (BMI) or waist-to-height ratio (WTH). Participants were excluded if they had any current/previous psychiatric history using the Structured Clinical Interview for DSM-IV Axis I Disorder Schedule, or if they took psychoactive medication or an eating disorder (measured with Eating Attitude Test > 20), food allergies, diabetes, smoking, or any contraindications to fMRI scanning. We also recorded the frequency, liking and craving for sugary and sweetened foods (Rolls and McCabe, 2007) e.g., “How frequently do you eat sugary foods?” with answers of either; a few times per month; 1–2 times per week; 3–4 times per week; or more than 5 times per week and “How frequently do you eat/drink foods with sweeteners?”, with answers of either; Never; Rarely; Sometimes; Often; Usually or Always. The Craving and Liking for sugary foods were scored as 1 for low and 10 for high craving on a Likert scale. All procedures comply with the ethical standards of the Helsinki Declaration of 1975, revised in 2013 and approval was obtained from the University of Reading Ethics committee, ethics ref: 2023–130-CM, all participants provided written informed consent.

### Pre-test 1 (Triangle test or Taste perception test)

Participants were entered into the study if they could distinguish 2 % sucrose from a control using a standard taste perception test (see supplementary doc).

### Pre-test 2 (Candy smell test retro nasal)

We used the candy smell test to check participants retro nasal olfactory performance (Renner, Mueller et al. 2009) (see supplemental doc).

### Pre-test (Smell test ortho nasal)

To check participants ortho nasal olfactory performance and to exclude anosmia we used the coffee smell test (Humphries and Singh, 2018) (see supplemental doc).

### Stimuli for the scan

The sucrose was >99.7 % pure with less than 0.04 % inverted sugar (i.e. fructose and glucose) and less than 0.06 % loss during drying, and sourced from Wiener Zucker, Feinkristallzucker, Austria and the sweet concentration of sucrose was 6 % (Wee, Tan et al. 2018). Sucrose was diluted and delivered in distilled water (6 g in 100 mL). A tasteless control solution (containing the main ionic components of saliva, 25 mM KCl + 2.5 mM NaHCO_3_) was used as a rinse condition on each trial.

### Nose clips

Soft plastic foam nose clips were used to block retro nasal smell (size approx. 2.7 ×1.6 in.) Frienda Ltd., China). The pleasantness, pain and comfort of the nose clips was piloted before the study, on 8 subjects. All participants rated the nose clip on a scale ranging between −4 and 4 for pleasure, pain and comfort, once at baseline and again after wearing the nose-clip for 4 min (the length of time they would be wearing the nose clip in each condition in the scanner).

To examine the effects of the nose clip on subjective ratings we used a repeated measures ANOVA with ratings (3 levels, pleasantess, pain and comfort) as one within subject factor and condition (2 levels, time1 and time2) as a second within subject factor. We found no main effect of ratings (F=0.16 (2,14) p = 0.85) or time (F=0.1 (1,7) p = 0.75) and no ratings * time interaction (F=2 (2,14) p = 0.17) (Table 1).

**Table 1:** Subjective ratings made before and after wearing nose clip.

**Baseline **			**After 4 min**		
**Mean (SD)**			**Mean (SD)**		
Pleas	Pain	Comfort	Pleas	Pain	Comfort
−0.16 (1.09)	−0.76 (1.90)	−0.26 (1.51)	−0.21 (1.41)	−0.42 (1.81)	−0.78 (1.69)

### Study design

The fMRI scans took place at the Centre of Integrative Neuroscience and Neurodynamics at the University of Reading. If scanned in the morning participants fasted overnight, if scanned in the afternoon they fasted for 3 h (no food, only water) before the scan. 10 participants had a morning scan, and 24 participants had an afternoon scan. 60–90 min before scanning all the participants were given a standardized meal similar to previous studies (a banana, a cup of orange juice, 2 crackers, ∼261 total calories) with the instruction to “eat until feeling comfortably full, without overeating” similar to our previous study (Thomas, Higgs et al. 2015). We asked participants to rate their hunger and mood, before the scan, on a visual analogue scale from 0 being not at all to 10 indicating the most ever felt. Subjects were screened for potential pregnancy and metal in their body before being placed in the fMRI scanner.

### Taste delivery

Tastes were delivered to the subject via separate long (∼3 m) thin Teflon tubes with a mouthpiece (∼ 1 cm in diameter) at one end, that was held by the subject comfortably between the centre of the lips. At the other end of the tubes were connected to separate reservoirs via syringes and one-way Syringe Activated Dual Check Valves (Model 14044–5, World Precision Instruments, Inc) which allowed any stimulus to be delivered manually by the researcher at exactly the right time indicated by the programme (Murray, Brouwer et al. 2014) thus avoiding the delays and technical issues experienced when using computerised syringe drivers.

### fMRI task

At the beginning of a trial, a white cross at the centre of the screen appeared for 2 s indicating the start. Then, sucrose was delivered in a 0.5 mL aliquot to the subject’s mouth, the green cross was presented at the same time on the visual display for 5 s. The instruction given to the subject was to move the tongue once as soon as a stimulus was delivered in order to distribute the solution round the mouth to activate receptors, and then to keep still until a red cross was shown, when the subject could swallow. Swallowing was 2 s, then the subject was asked to rate the ‘pleasantness’ (+2 to –2) to measure hedonic value, and asked to rate the mouth fullness (richness) of the taste in their mouth (0 to +4) to measure the sensory intensity of sucrose, on a visual analogue scale by moving a bar to the appropriate point on the scale using a button box, ratings similar to those used in previous taste/fmri studies (Rolls, 2019). Each rating period was 5 s long. After the last rating on each trial 0.5 mL of the tasteless control solution was administered in the same way as the sucrose stimulus at the same time as a green cross was presented on the visual display for 5 s. The control tasteless rinse was used as the comparison condition to allow somatosensory effects produced by liquid in the mouth, and the single tongue movement made to distribute the liquid throughout the mouth, to be subtracted in analysis (O'Doherty et al., 2001, De Araujo et al., 2003). The control taste was not subjectively rated. Then, a grey cross was presented for a duration between 0.8 s and 2 s (jittered) to indicate the end of the trial. Then the screen was black for 2 s before a new trial started. Each trial lasted ∼30 sec. Using a block design there were 7 trials of sucrose and control condition with the nose clip off. Then the scanner was stopped ∼7–10 min, and the participant had a break before the nose clip was placed on the nose. During the break participants were told to let go of the taste tubes and just relax and they could close their eyes. Although we have shown previously no habituation effects in the subjective pleasantness and mouth fullness of sucrose after 10 presentations over the course of a 30 min task (please add a to this ref) we also introduced a break between the blocks in this study to avoid habituation effects and time to introduce the nose clip. After the break we ran another localiser scan followed by 7 trials of sucrose taste and control condition with nose clip on. The whole task took ∼30 min, including stopping and starting the scanner.

### fMRI data acquisition

Blood oxygenation level dependent (BOLD) functional MRI images were acquired using a three-Tesla Siemens scanner (Siemens AG, Erlangen, Germany) with a 32-channel head coil. During the task, around 1500 volumes were obtained for each participant, using a multiband sequence with GRAPPA and an acceleration factor of 6. Other sequence parameters included a repetition time (TR) of 700 ms, an echo time (TE) of 30 ms, and a flip angle (FA) of 90°. The field of view (FOV) covered the whole brain with a voxel resolution of 2.4 × 2.4 × 2.4 mm^3^. Moreover, structural T1-weighted images were acquired utilizing a magnetization prepared rapid acquisition gradient echo sequence (TR = 2020 ms, TE = 3.02 ms, FA = 9°) with a FOV covering the whole brain and a voxel resolution of 1 × 1×1 mm^3^.

### fMRI data analysis

The imaging data were analysed using SPM12 and pre-processed with realignment, coregistration, segmentation, normalization to the MNI coordinate system (Montreal Neurological Institute; Collins et al., 1994) and smoothed with a 6 mm full width at half maximum isotropic Gaussian kernel. The time series at each voxel was low-pass filtered with a haemodynamic response kernel and non-sphericity was estimated and corrected for, with a high-pass filter cut-off period of 128 s.

In the single-event design, a general linear model was then applied to the time course of activation in which stimulus onsets were modelled as single impulse response functions and then convolved with the canonical hemodynamic response function. Linear contrasts were defined to test specific effects. Time derivatives were included in the basis functions set. Following smoothness estimation, linear contrasts of parameter estimates were defined to test the specific effects of each condition with each individual dataset. Voxel values for each contrast resulted in a statistical parametric map of the corresponding t statistic (transformed into the unit normal distribution (SPM z)). Movement parameters and were added as additional regressors.

At the second level, we report the main effects of sucrose with nose clip off vs the corresponding control tasteless conditions with nose clip off (supplemental data), and sucrose with nose clip on vs sucrose with nose clip off, thresholded at p < 0.05 corrected (familywise-error (FWE) and p values cluster corrected at both p < 0.05 False Discovery Rate (FDR) and p < 0.05 FWE. We also added gender, hunger level and scan time as covariates of no interest.

We then examined regions of interest (ROI) spheres (10 mm) for the anterior insula (primary taste cortex, [-32, 16, 2]) posterior insula [-38, -2, -12] and postcentral gyrus [60, −16, 24] using WFU pickatlas, identified in the meta-analysis on sweet tastes in humans (Roberts, Giesbrecht et al. 2020). We examined the olfactory regions; the piriform cortex, olfactory cortex and the orbitofrontal cortex using aal atlas anatomical masks in WFU pickatlas. Given our interest in retro nasal effects (Small et al., 2005) and attention to odors (Veldhuizen and Small, 2011) we also created a sphere (10 mm) in the pgACC [3, 42, -9] (Small et al., 2005) and examined anatomical masks of the mOFC (Small et al., 2005) and sgACC (BA25) (Veldhuizen and Small, 2011) using aal atlas in WFU pickatlas. Finally, as we are interested in the retro nasal contribution to the rewarding effects of taste we examined the nucleus accumbens (Berridge, 2009) and amygdala (Gottfried et al. 2003) using (IBASPM71 atlas) and aal atlas anatomical masks, respectively, in WFU pickatlas. Data were extracted using the SPM ROI analysis Matlab code and MarsBar and analysed with paired-sample t tests in excel. We also examined correlations between the ROI data and the subjective ratings.

We also examined if the nose clip effected the time to peak activity. We calculated the time to maximum peak activity within the first 10 s after the onset of sucrose delivery from the time course data, using the max function in Matlab.

## Results

### Demographic data for fMRI study

34 participants took part with a mean age of 25 yrs. See Table 2 for demographics.

**Table 2:** Demographics.

	**All (n = 34) Mean score (SD)**
Age, years	25.71 (8.25)
Gender, female/male: *n*	24/10
Body mass index	22.00 (2.68)
Eating Attitudes Test	3.09 (3.20)
Craving for sugary foods	5.11 (1.99)
Liking for sugary foods	5.85 (1.98)
Freq eating sugary foods	3.44 (2.09)
Freq eating/drinking foods with sweeteners	3.97 (2.11)

### Pre-test results of sensitivity to 2 % sucrose

Twenty-one participants passed the pre-test with 6 out of 6 trials correct the first time. Ten participants passed the pre-test with 5 out of 6 trials correct the first time and three participants got 6 of the 6 trials correct on their second attempt, so were also included in the study.

### Pre-test candy smell test

With the nose clip off participants identified the flavours with average accuracy of 84 % (± 14). For nose clip on accuracy dropped to 31 % (± 20) similar to previous studies (Renner, Mueller et al. 2009).

### Pre-test (Smell test ortho nasal)

All participants identified the coffee compared to no coffee and rated the coffee as above average intensity and higher (6.70 ± 1.78) than the empty cup intensity (1.17 ± 1.90), (t(22) = 12.04, p < 0.001). The intensity of the coffee smell was higher with the nose clip off (6.70 ± 1.78) than with the clip on (0.26 ± 0.59), (t(22) = 16.7, p < 0.001).

## fMRI scan day

### Subjective hunger and mood

Participants had relatively high mood and low hunger levels before the scan (Table 3).

**Table 3:** Visual Analogue Scale of Mood and Appetitie.

	**Mean score (**± **SD)**
How hungry do you feel right now? How full do you feel right now?	4.35 ± 2.30 4.05 ± 2.11
Alertness	6.08 ± 2.40
Disgust	0.91 ± 1.23
Drowsiness	3.05 ± 2.66
Anxiety	1.79 ± 1.55
Happiness	6.11 ± 1.93
Nausea	0.70 ± 0.97
Sadness	0.55 ± 1.05
Withdrawn	1.08 ± 1.76
Faint	1.08 ± 1.84

Rate between 0 and 10, where 0 = Not at all, 10 = Most ever felt

### Pleasantness and fullness ratings

To check for habituation effects we examined the ratings at the beginning and the end of block 1 i.e., trial 1 vs trial 7 in the nose clip off condition. Using paired samples *t*-test we found no differences in pleasantness t(32) = 0.65, p = 0.51, or mouth fullness t(31) = 1.88, p = 0.07 indicating no habituation to sucrose taste across trials and between the blocks.

**Fig. 1:**
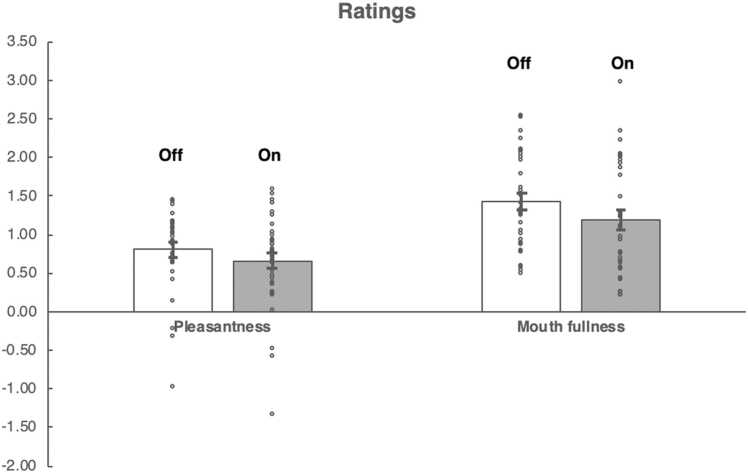
Pleasantness and Mouth Fullness ratings for sucrose nose clip on and nose clip off conditions in the scanner.

To examine the effects of the nose clip on subjective ratings we used a repeated measures ANOVA with ratings (2 levels, pleasantness, mouth fullness) as one within subject factor and condition (2 levels, nose clip on, nose clip off) as a second within subject factor. We found a main effect of ratings (F=27.5 (1,33) p < 0.001) and a main effect of condition (F=6.6 (1,33) p = 0.015) but no ratings * condition interaction (F=0.39 (1,33) p = 0.54) (Fig. 1). Follow up paired sample *t*-tests showed that mouth fullness was rated higher for nose clip off than nose clip on t(33) = 2.5, p = 0.017.

### ROI analysis

**Fig. 2:**
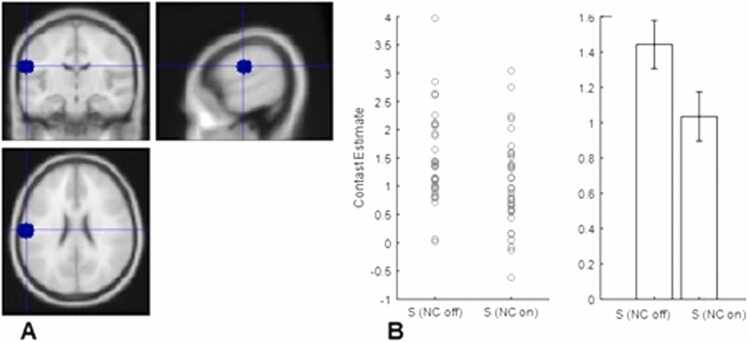
A. Left postcentral gyrus ROI. B. Contrast estimates extracted from ROI using marsbar for sucrose nose clip off and nose clip on conditions, error bars SEM.

**Fig. 3:**
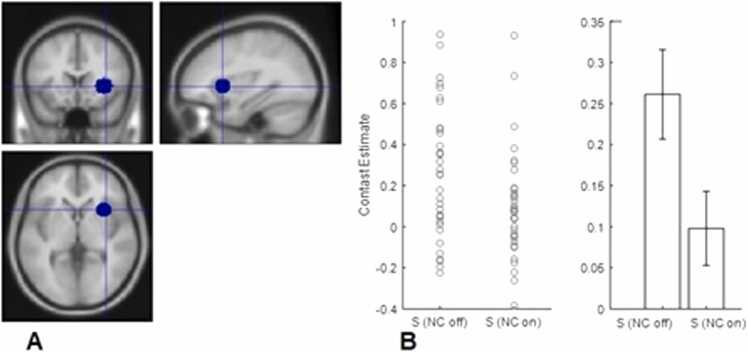
A. Right anterior Insula ROI. B. Contrast estimates extracted from ROI using marsbar for sucrose nose clip off and nose clip on conditions, error bars SEM.

We found greater neural activity in the sucrose nose clip off vs on in the left (Fig. 2) and right postcentral gyrus, right anterior (Fig. 3) and right posterior insula, the olfactory cortex (Figure S1), piriform cortex, sgACC (Figure S2) right NAcc (Figure S3) activity survived when controlling for multiple comparisons (Table 4).

**Fig. 4:**
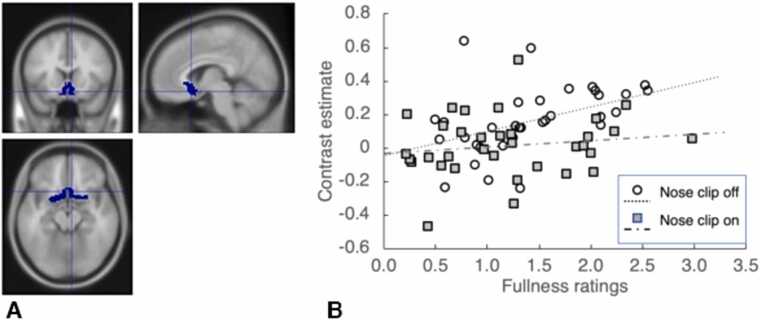
A. Olfactory cortex ROI. B. Correlations between mouth fullness ratings and brain activity to sucrose with the nose clip on and nose clip off conditions.

**Table 4:** Sucrose Nose clip off vs. Nose clip on.

**ROI**	*t value*	*p value*	*cohens D*			
mOFC	1.96	0.03	0.34			
pgACC	1.11	0.14	0.19			
sgACC	3.08	0.002*	0.53			
Olfactory	3.89	0.0002*	0.67			
Piriform	3.04	0.002*	0.52			
	***Left***			***Right***		
	*t value*	*p value*	*cohens D*	*t value*	*p value*	*cohens D*
Postcentral gyrus	3.54	0.0006*	0.61	3.1	0.002*	0.53
Anterior Insula	2.6	0.007	0.45	2.88	0.003*	0.49
Posterior Insula	1.2	0.12	0.21	2.93	0.003*	0.50
NAcc	2.07	0.02	0.38	2.93	0.003*	0.52
Amygdala	1.98	0.03	0.34	2.68	0.005	0.46
OFC	2.09	0.02	0.36	1.36	0.09	0.23

* Survives correction for multiple comparisons, (0.05/17 ROIs, p = 0.003)

### Temporal effects

We examined the time to peak activity after the sucrose taste in the nose clip on vs off conditions. Using repeated measures ANOVA with ROIs as one within subject factor and condition (nose clip on/off) as a second within subject factor. We found a main effect of ROI (F=12.6 (1,8) p < 0.001) but no main effect of condition and no ROI * condition interaction (Figure S4).

### Parametric modulation

We found positive correlations between ROIs and mouth fullness ratings in olfactory cortex (rho = 0.44, p = 0.01) (Fig. 4), the sgACC (rho = 0.34, p = 0.046), the pgACC (rho = 0.37, p = 0.03) and the mOFC (rho = 0.36, p = 0.036) for nose clip off, but these did not survive correction for multiple comparisons. No correlations between ratings and ROI data were found for the nose clip on.

### Exploratory whole brain analyses

#### Main effects of taste stimuli

The sucrose vs the control activated regions such as the primary taste cortex (insula), primary somatosensory cortex (postcentral gyrus), and the precentral gyrus and caudate (Table S1). There were no significant activations for the opposite contrast, control vs sucrose.

#### Nose clip off vs on

When examining the whole brain results (Table S3) we found reduced activity for the contrast sucrose nose clip off vs on in regions such as the rolandic operculum, precuneus and post central gyrus, these results were apparent only when using a p = 0.001 uncorrected threshold. There were no regions activated under the opposite contrast, at any threshold. There was reduced precuneus activity for the contrast tasteless control clip off vs on, but only at p = 0.001 uncorrected threshold. There were no regions activated under the opposite contrast, at any threshold.

## Discussion

This is the first study to examine the subjective and neural effects of retro nasal occlusion with a nose clip while tasting sucrose. We provide novel evidence of reduced subjective mouth fullness consistent with previous behavioural findings of reduced sucrose sweetness with retro nasal occlusion (Mu et al., 2024, Yang et al., 2024). We also provide first evidence that the postcentral gyrus, part of the somatosensory cortex was reduced with the nose clip on. Decreased postcentral gyrus activity could reflect an ‘objective’ decreased sensing of sweetness from sucrose as the postcentral gyrus has been found previously activated by sweet tastes (Yeung and Wong, 2020) and is part of the somatosensory cortex (Small, 2012) and is modulated by sweet taste intensity (van Meer et al. 2023). We have also previously found greater postcentral gyrus activity to sucrose vs. sucralose (please add a to this ref) and vs. stevia (Ko et al., 2025b) and greater postcentral gyrus activity with the addition of flavour modifiers to sweeteners (Ko et al., 2025b, please add a to this ref). The implications therefore are that modulation of the postcentral gyrus could make foods more sucrose like and this could be via retronasal pathway contributions.

We also found reduced anterior and posterior insula (Dalenberg, Hoogeveen et al. 2015, Roberts, Giesbrecht et al. 2020) olfactory cortex and piriform cortex activity reduced to sucrose with a nose clip on.

Further, neural activity tracked subjective mouth fullness but only with the nose clip off, not on. Taken together, our results imply that retro nasal pathways contributor to the perceptual processing of “non-volatile” substances (He et al., 2023, He et al., 2024).

The secondary olfactory areas (OFC) were less impacted by retro nasal occlusion perhaps because this multimodal region is much less dependent on signals coming purely from one modality (Rolls, 2019) and therefore are still activated by the taste in the mouth even with the nose clip on. This fits with previous work where a nose clip affected taste and aroma processing but no other auditory or visual senses (Yang, Kim et al. 2024).

Retro nasal occlusion reduced sgACC neural activity, this could reflect the participants difficulty attending to the tastes in order to rate them (Sabri et al., 2005, Veldhuizen and Small, 2011) and is consistent with our finding that the sgACC, olfactory cortex and neighbouring pgACC and mOFC ROIs tracked mouth fullness but only when the nose clip was off. Prefrontal cortex multi-modal regions show greater activation to tastes when combined with savory odours than to the sum of the activations by the taste and olfactory components presented separately (McCabe and Rolls, 2007, Rolls, 2019). This could suggest that the nose clip reduces the integration of taste and olfactory components making it more difficult to perceive sucrose.

When examining the effects of retro nasal occlusion we also found reduced activity in the NAcc, a hub related to feeding, homeostatic and hedonic circuits, that facilitates behaviour via its downstream projections (Marinescu and Labouesse, 2024). The ventral striatum is at the crossroads of olfactory and reward pathways and receives direct projections from the primary olfactory cortex (Ubeda-Bañon, Novejarque et al. 2007) and the dopaminergic midbrain (Ikemoto, 2007) and is greatly involved in odour-guided eating behaviour (Murata, 2020). Hence reduced activity in this region supports the idea that potential retro nasal olfactory signals from the sucrose taste are being occluded.

Examining the exploratory whole brain results we found that the retro nasal occlusion reduced neural activity to sucrose taste in the rolandic operculum (RO) and precuneus. The rolandic operculum plays a central role in flavour percept formation (Small, Voss et al. 2004) and neural taste and smell signals are integrated here (Suen, Yeung et al. 2021).The operculum, is a large structure with three lobes and a complex array of functions including sensory, motor, autonomic and cognitive processing. In humans, these are extended with the addition of language (Mălîia, Donos et al. 2018). Studies mapping the function of the RO, using direct electrical stimulation, find it involved in oropharyngeal responses with the most widespread and common mapping it to the pharynx–larynx or the tongue (Mălîia, Donos et al. 2018). Further when stimulated participants report experiencing taste, making the RO a likely candidate for the primary gustatory cortex (Mălîia, Donos et al. 2018). Connections between the RO and the insula support its role in feeding behaviour while connections with the frontal operculum, premotor area, fusiform gyrus and post central gyrus support its role in speech production (Mălîia, Donos et al. 2018). Due to such connections, some suggest a link between flavour perception and language development, citing gustation-language connectivity and chimpanzees' vocal food communications (Schel et al., 2013, Kalan et al., 2015).

Finally, we also found reduced precuneus activity to sucrose taste with the nose clip on. The precuneus is primarily involved in complex cognitive functions like episodic memory retrieval, self-processing, visuo-spatial imagery, and imagining future events, essentially acting as a hub for integrating personal experiences and constructing mental scenarios; it is considered a core part of the brain's "default mode network" which is active during resting states and internal thought processes (Cavanna and Trimble, 2006). Therefore, reduced activity in this region to sucrose (and to the control) with the nasal occlusion could reflect a difficulty in determining the percept of the stimulus and a need to recruit taste memories.

Taken together, we provide neuroscientific evidence that retro nasal sensations are playing a role in sucrose perception. Knowing this could help explain how olfactory impairments (e.g., aging, illness, or COVID-19) impact appetite and altered eating behaviours. Further research could examine how retro-nasal occlusion effects other sweet tastes such as non-nutrient sweeteners like stevia. As they may not be detected via retro nasal pathways to the same degree which could have meaningful implications for low-sugar food creation.

Further, our previous work (Ko et al., 2025b, please add a to this ref ) found that flavour modifiers combined with sweeteners could activate regions like the postcentral gyrus i.e. make a non-nutrient sweetener more like sucrose. As the current findings show that blocking retro nasal pathways reduces activity in regions like the post central gyrus, it is possible that non-nutrient sweetened foods could be made more acceptable i.e. more sugar-like, via retro nasal pathway aroma modulation. Thus, this study also contributes to a broader understanding of how retro nasal pathway activation could help manufacturers create sugar-free or low sugar foods with improved taste. This could support public health goals without compromising enjoyment. Understanding neural responses to sucrose and the contribution of retro nasal pathways therefore not only provides novel scientific evidence for the role of retro nasal pathways in non-volatile substances but also offers a roadmap for enhancing the hedonic and health aspects of sweetened foods.

## CRediT authorship contribution statement

**Ciara McCabe:** Conceptualization, Formal analysis, Funding acquisition, Methodology, Project administration, Supervision, Writing – original draft, Writing – review & editing. **Hee-kyoung Ko:** Conceptualization, Data curation, Formal analysis, Investigation, Methodology, Visualization, Writing – original draft, Writing – review & editing. **Jingang Shi:** Conceptualization, Funding acquisition, Methodology, Resources, Writing – review & editing. **Eidenberger Thomas:** Conceptualization, Data curation, Formal analysis, Methodology, Writing – review & editing. **Weiyao Shi:** Conceptualization, Funding acquisition, Methodology, Writing – review & editing.

## Author contributions

CMcC, TE, JS, WS conceived and designed research. JS, WS and TE supplied the sucrose. HK and TE conducted research and analysed the data supported by CMcC. CMcC and HK drafted the article. All authors actively participated in editing and reviewing the manuscript.

## Data accessibility statement

The data that support the findings of this study are available from the corresponding author, upon reasonable request.

## Declaration of Competing Interest

The authors declare the following financial interests/personal relationships which may be considered as potential competing interest. Weiyao Shi and Jingang Shi are employees of EPC Natural Products Co., Ltd . The work was conducted independently at the NRG laboratory of Prof. McCabe at The University of Reading solely for the purpose of scientific understanding. All authors declare that they have no other known competing financial interests or personal relationships that could have appeared to influence the findings reported in this paper.

## Footnotes

Appendix A Supplementary data associated with this article can be found in the online version at doi:10.1016/j.ibneur.2025.10.020.
