# Supplementary material for: Retro nasal blockade reduces the neural processing of sucrose in the human brain
Source: IBRO Neurosci Rep. 2025 Oct 31;19:886–93. doi: 10.1016/j.ibneur.2025.10.020 (PMC12834034; doi:10.1016/j.ibneur.2025.10.020)
Supplement: Supplementary file 1 — Supplementary material [file mmc1.docx]

*Supplementary doc*

***Retro Nasal blockade reduces the Neural Processing of Sucrose in the Human Brain***

*Pre-test 1 (Triangle test or Taste perception test)*

The 34 participants were entered into the study if they could distinguish 2% sucrose from a control using a standard taste perception test as follows: The participants were randomly allocated to the following sequences of two samples A (distilled water) and B (20 g sucrose/litre [2 % Sucrose]): ABB, AAB, ABA, BBA, BAA and BAB. For the individual performance, each participant received all six sequences in random order. In a sequence, the participants took the whole 10 mL of each sample into their mouth, swirled and coat the solution around their mouth for 3 seconds and then spit it into a spittoon. On each trial after tasting all three, they indicated which was different from the other two. Participants who yielded correct identification of at least 5 out of the 6 trials on a second attempt, were recruited to the study.

*Pre-test 2 (Candy smell test retro nasal)*

We used the candy smell test to check participants retro nasal olfactory performance (26). This test examines participants ability to identify the flavour of a candy (500 mg) placed on the middle of the tongue from 6 possible choices (6-alternative, forced-choice procedure) strawberry, banana, orange, coffee, cherry, or pineapple. Participants can suck the candy or chew it if necessary. The participants wrote down one of the choices. If they cannot identify the candy they can skip to the next trial. Each participant performed 5 trials with nose clip on and 5 trials with nose clip off. Between trials participants rinse their mouths with water. There was no feedback to the participants whether their responses were correct or incorrect. We expected less than 40 - 50% correct (1 or 2 corrects / 5 trials) for nose clip on condition and 80-100% correct (4 or 5 corrects / 5 trials) for nose clip off condition in line with previous studies (26).

*Pre-test (Smell test ortho nasal)*

To check participants ortho nasal olfactory performance and to exclude anosmia we used the coffee smell test (27) reported to have excellent validity with sensitivity of 93% and specificity of 96% in comparison to a 12 item Sniffin Sticks test kit (28).  We prepared a 100ml cup with grounded coffee beans and one empty cup. In a trial, the participants were asked to close their eyes and sniff from a cup that was presented to them (either coffee or empty) they had to report the smell by marking on 0 – 10 scales the smell intensity. 0 indicated no smell at all with 10 indicating a very strong smell. Each participant performed 5 trials with nose clip on and 5 trials with nose clip off.

*Figure S1*


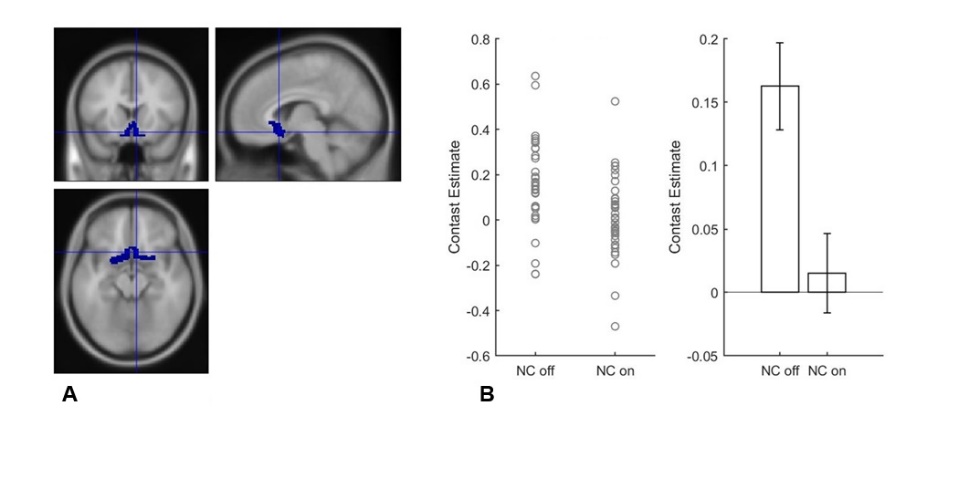


**Figure S1.** A. Olfactory Cortex ROI. B. Contrast estimates extracted from ROI using marsbar for sucrose nose clip off and nose clip on conditions, error bars SEM.

*Figure S2*


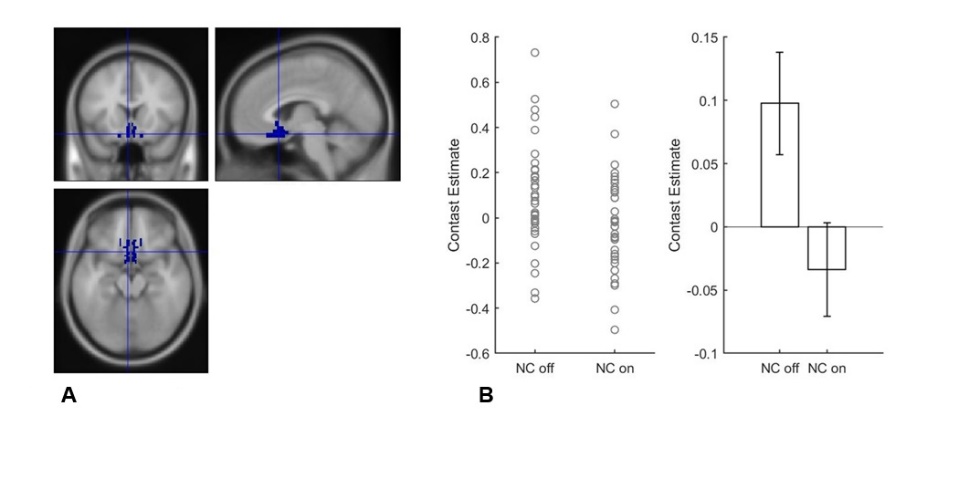


**Figure S2.** A. sgACC (BA25) ROI. B. Contrast estimates extracted from ROI using marsbar for sucrose nose clip off and nose clip on conditions, error bars SEM.

*Figure S3*


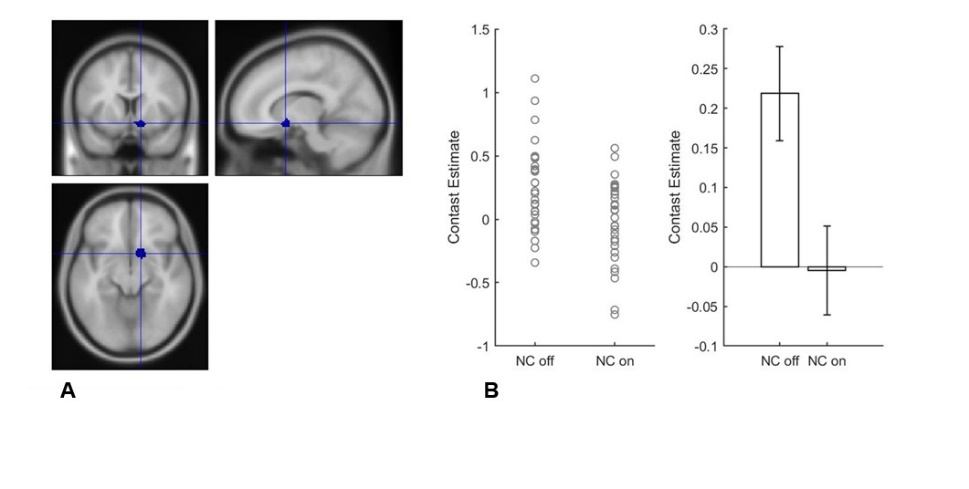


**Figure S3.** A. Right NAcc ROI. B. Contrast estimates extracted from ROI using marsbar for sucrose nose clip off and nose clip on conditions, error bars SEM.

*Figure S4*


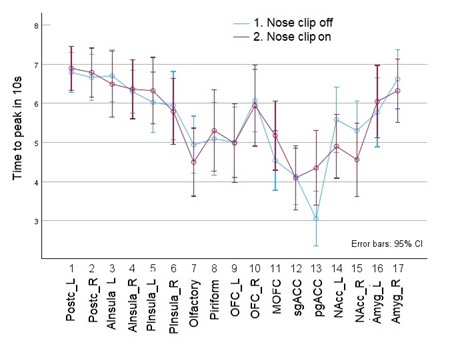


**Figure S4.** Time to first peak (10 s time bin) for each ROI and for each condition: nose clip on and nose clip off.

*Figure S5*

*Main effects of taste stimuli*

**Table S1**

| **Sucrose - control (Nose Clip Off)**  Threshold: p=0.05 FWE corrected | | | | | | | |
| --- | --- | --- | --- | --- | --- | --- | --- |
| **Region** | **x(mm)** | **y(mm)** | **z(mm)** | **Z-score** | **voxels** | **p(FWE-corr)** | **p(FDR-corr)** |
| Postcentral | -42 | -18 | 52 | 6.314 | 152 | < 0.0001 | < 0.0001 |
| Precentral | -35 | -23 | 55 | 6.022 |  |  |  |
| Supp Motor Area | 4 | 10 | 55 | 6.059 | 252 | 0 | < 0.0001 |
| Supp Motor Area | -6 | 15 | 48 | 5.877 |  |  |  |
| Insula | 37 | 20 | 7 | 5.704 | 106 | < 0.0001 | < 0.0001 |
| Mid Cingulate gyrus | -8 | 25 | 31 | 5.466 | 10 | =0.000863 | =0.050467 |
| Hippocampus | 42 | -30 | -8 | 5.400 | 22 | < 0.0001 | =0.00449 |
| Insula | -35 | 15 | 7 | 5.260 | 29 | < 0.0001 | =0.001665 |
| Caudate | 18 | 15 | 7 | 5.131 | 23 | < 0.0001 | =0.004377 |
| Insula | -42 | 18 | 0 | 5.131 | 10 | 0.000863 | 0.050467 |
| Threshold: 0.0001 uncorrected | | | | | | | |
| **Region** | x(mm) | y(mm) | z(mm) | Z-score | voxels | p(FWE-corr) | p(FDR-corr) |
| Superior frontal gyrus | -23 | -4 | 55 | 5.082 | 518 | < 0.0001 | < 0.0001 |
| Inferior parietal | -54 | -23 | 48 | 3.736 |  |  |  |
| Insula | 37 | 20 | 7 | 5.704 | 1185 | 0 | < 0.0001 |
| Putamen | 23 | 15 | -8 | 5.244 |  |  |  |
| Frontal operculum | 47 | 3 | 24 | 4.822 |  |  |  |
| Hippocampus | 42 | -30 | -8 | 5.400 | 160 | < 0.0001 | < 0.0001 |
| Superior temporal gyrus | 37 | -33 | 7 | 4.938 |  |  |  |
| Cerebellum | 20 | -59 | -22 | 4.943 | 28 | =0.02442 | =0.049727 |
| Insula/Rolandic operculum | 37 | -4 | 14 | 4.911 | 31 | =0.018074 | =0.039507 |
| Superior parietal | -28 | -57 | 57 | 4.894 | 74 | =0.000476 | =0.001917 |
| Precuneus | -13 | -59 | 64 | 3.787 |  |  |  |
| Mid frontal cortex | 40 | 34 | 19 | 4.561 | 39 | =0.008436 | =0.01988 |

**Table S2**

| **Sucrose - control (Nose Clip On)**  Threshold: p=0.05 FWE corrected | | | | | | | |
| --- | --- | --- | --- | --- | --- | --- | --- |
| **Region** | **x(mm)** | **y(mm)** | **z(mm)** | **Z-score** | **voxels** | **p(FWE-corr)** | **p(FDR-corr)** |
| Supp Motor Area | 4 | 20 | 48 | 6.565 | 277 | 0 | < 0.0001 |
| Supp Motor Area | -6 | 13 | 50 | 6.010 |  |  |  |
| Caudate | -6 | 10 | 4 | 6.237 | 199 | < 0.0001 | < 0.0001 |
| Caudate | 11 | 15 | 7 | 6.202 |  |  |  |
| Postcentral gyrus | -37 | -21 | 52 | 6.390 |  |  |  |
| Precentral gyrus | -37 | -26 | 60 | 6.296 | 304 | 0 | < 0.0001 |
| Precentral gyrus | 52 | 6 | 33 | 5.708 | 40 | < 0.0001 | =0.000138 |
| Insula | 32 | 18 | 7 | 5.044 | 32 | < 0.0001 | =0.000486 |
| Inferior frontal gyrus | -40 | 25 | 26 | 5.229 | 17 | =0.000142 | =0.007097 |
| Inferior frontal operculum | -47 | 22 | 31 | 5.202 |  |  |  |
| Insula | -30 | 25 | 9 | 5.225 | 22 | < 0.0001 | =0.00264 |
| Threshold: 0.0001 uncorrected | | | | | | | |
| Region | x(mm) | y(mm) | z(mm) | Z-score | Num of voxel | p(FWE-corr) | p(FDR-corr) |
| Mid cingulate gyrus | 11 | 30 | 31 | 4.590 | 1022 | 0 | < 0.0001 |
| Superior anterior cingulate | -8 | 25 | 28 | 4.335 |  |  |  |
| Medial superior frontal gyrus | 11 | 32 | 43 | 4.048 |  |  |  |
| Superior parietal | 30 | -59 | 57 | 4.012 | 282 | < 0.0001 | < 0.0001 |
| Inferior parietal | 40 | -54 | 50 | 4.722 |  |  |  |
| Precuneus | 6 | -74 | 43 | 4.367 | 72 | =0.000518 | =0.002019 |
| Mid Occipital | -28 | -90 | 14 | 4.246 | 22 | =0.04496 | =0.09369 |

**Table S3**

| **Table S3:**   **Sucrose**  **(Nose Clip Off – Nose Clip On)**  *Threshold: p=0.001 uncorrected* | | | | | | | |
| --- | --- | --- | --- | --- | --- | --- | --- |
| Region | x(mm) | y(mm) | z(mm) | Z-score | Num of voxel | p(FWE-corr) | p(FDR-corr) |
| Rolandic operculum | 40 | 1 | 16 | 5.07 | 71 | =0.036 | =0.060 |
| Precuneus | -1 | -83 | 43 | 4.82 | 143 | =0.001 | =0.003 |
| Lingual gyrus | -16 | -64 | 0 | 4.18 | 227 | < 0.0001 | <0.001 |
| Postcentral gyrus | -61 | -26 | 24 | 4.08 | 146 | =0.001 | =0.003 |
| Rolandic operculum | 56 | 3 | 7 | 4.06 | 91 | =0.013 | =0.026 |
